# Porcine Deltacoronavirus Nucleocapsid Protein Suppressed IFN-β Production by Interfering Porcine RIG-I dsRNA-Binding and K63-Linked Polyubiquitination

**DOI:** 10.3389/fimmu.2019.01024

**Published:** 2019-05-09

**Authors:** Ji Likai, Li Shasha, Zhu Wenxian, Ma Jingjiao, Sun Jianhe, Wang Hengan, Yan Yaxian

**Affiliations:** Shanghai Key Laboratory of Veterinary Biotechnology, School of Agriculture and Biology, Shanghai Jiao Tong University, Shanghai, China

**Keywords:** PDCoV, nucleocapsid protein, porcine RIG-I, IFN-β, ubiquitination

## Abstract

Porcine deltacoronavirus (PDCoV) is a newly detected porcine coronavirus causing serious vomiting and diarrhea in piglets, especially newborn piglets. There has been an outbreak of PDCoV in worldwide since 2014, causing significant economic losses in the pig industry. The interferon (IFN)-mediated antiviral response is an important component of virus-host interactions and plays an essential role in inhibiting virus infection. However, the mechanism of PDCoV escaping the porcine immune surveillance is unclear. In the present study, we demonstrated that the PDCoV nucleocapsid (N) protein antagonizes porcine IFN-β production after vesicular stomatitis virus (VSV) infection or poly(I:C) stimulation. PDCoV N protein also suppressed the activation of porcine IFN-β promoter when it was stimulated by porcine RLR signaling molecules. PDCoV N protein targeted porcine retinoic acid-inducible gene I (pRIG-I) and porcine TNF receptor associated factor 3 (pTRAF3) by directly interacting with them. The N-terminal region (1–246 aa) of PDCoV N protein was important for interacting with pRIG-I and interfere its function. We confirmed that PDCoV N antagonizes IFN-β production by associating with pRIG-I to impede it from binding double-stranded RNA. Furthermore, porcine Riplet (pRiplet) was an important activator for pRIG-I by mediating the K63-linked polyubiquitination. However, PDCoV N protein restrained the pRiplet binding pRIG-I to inhibit pRIG-I K63-linked polyubiquitination. Taken together, our results revealed a novel mechanism by which PDCoV N protein interferes with the early activation of pRIG-I in the host antiviral response. The novel findings provide a new insight into PDCoV on evading the host innate immune response and may provide new therapeutic targets and more efficacious vaccines strategies for PDCoV infections.

## Introduction

Porcine deltacoronavirus (PDCoV), a newly detected porcine coronavirus, as well as porcine transmissible gastroenteritis virus (TGEV), porcine rotavirus (PRV), and porcine epidemic diarrhea virus (PEDV) are the major pathogens of the porcine epidemic diarrhea disease. They cause microscopic intestinal lesions leading to serious diarrhea and often dehydration to death (1, 2). PDCoV as a pathogen was identified in 2012 in Hong Kong (3). Until now PDCoV strains have been isolated from a few regions and countries, including the United States, China, South Korea, Laos, and Thailand (4–7). PDCoV is an enveloped, single-stranded, positive-sense RNA virus with the genome length of approximately 25 kb. The genome arrangements are in the order of 5′ untranslated region (UTR), open reading frame 1a/1b (ORF1a/1b), spike (S), envelope (E), membrane (M), accessory protein 6 (NS6), nucleocapsid (N), accessory protein 7 (NS7), accessory protein 7a (NS7a), and 3′ UTR (8, 9). PDCoV has only been found infectious in swine until now. However, PDCoV could use the aminopeptidase N (APN) of mammalian and avian species to efficiently infect cells of an unusual abroad species range, including humans and chickens (10).

The host innate immune response serves as the first line of defense to resist pathogenic microorganism infection and replication. The viral pathogen is sensed by pattern recognition receptors (PRRs) of the infected host cells to induce an antiviral response. The retinoic acid-inducible gene-1 (RIG-I)-like receptors (RLRs) are a major member of host PRRs, including three homologous protein, RIG-I, melanoma differentiation associated gene 5 (MDA5), and DExH-box helicase 58 (DHX58/LGP2) (11). RIG-I and MDA5 were the activators of interferon production post RNA virus infection or the double-stranded RNA (dsRNA) analog polyinosine and polycytidilic acid (poly(I:C)) (12). RIG-I also resides in an autorepression state in normal physiological status cells by covering its N-terminal tandem caspase activation and recruitment domains (CARDs), which are necessary for interaction with mitochondrial antiviral signaling protein (MAVS, also known as IPS-1/VISA/Cardif) (13–15). The Riplet, also known as ring finger protein 135 (RNF135), is a necessary E3 ligase for releasing RIG-I autorepression by K63-linked polyubiquitination (16). The tripartite motif containing 25 (TRIM25) is another key E3 ligase for RIG-I activation by mediating the K63-linked polyubiquitination of RIG-I N-terminal CARDs (17). However, MDA5 does not adopt an auto-repression state in the ligand-free state (11). MDA5 also interacts with MAVS via CARDs to active the type I interferon signaling pathway. The MAVS as the polymeric signaling scaffold could recruit and activate the signaling proteins including tumor necrosis factor receptor associated factor 2 (TRAF2), TRAF3, TRAF5, TRAF6, and associated serine kinases (TBK1 and the IKK family) (18, 19). The activation of TBK1 could activate master transcription factors IRF3 and NF-κB translocation into the cell nucleus, and then induce the antiviral genes' production, including IFN-β and major antiviral cytokines (20). Type I IFNs are secreted and bind to the cell surface receptors of both virus-infected and non-infected neighbor cells to induce interferon-stimulating genes (ISGs) for antivirals by activating the JAK-STAT pathway.

However, viruses take on a variety of tactics to escape the host innate immune surveillance during the infection and replication. AIV NS1 protein also could both interact with human TRIM25 and Riplet to suppress RIG-I activation (21). NS1 proteins from human but not swine or avian influenza virus strain were able to interact with human Riplet in a species-specific manner (22). West Nile virus NS1 also antagonizes IFN-β production by inhibiting RIG-I and MDA5 K63-linked polyubiquitination (22). Coronavirus nucleocapsid proteins play the most fundamental role in packaging the viral genome and viral assembly with a similar topological structure (23). When the coronavirus infects the host cells, the N protein is also abundantly produced to regulate the host cell cycle, cell stress responses, immune system interference, and signal transduction (24). Severe acute respiratory syndromes (SARS) and middle east respiratory syndrome (MERS) coronavirus N protein could counteract with human TRIM25 to suppress RIG-I activation (25). PEDV N protein suppressed IFN-β and NF-κB activation by sequestering the formation of human TBK1 and IRF3 complex in HEK293T cells (26). However, PEDV N protein could activate the NF-κB and up-regulate the IL-8 expression in porcine intestinal epithelial cell (IEC), which is the target host cell of PEDV (27, 28). The other important porcine coronavirus, TGEV infection could prominently promote NF-κB activation and IFN-β production through RLR signaling in PK-15 cells (29). PDCoV infection could suppress porcine RIG-I-mediated IFN-β production in LLC-PK1 cells (30). PDCoV Nsp5 and NS6 were confirmed as the important regulatory viral proteins to inhibit the IFN-β production (31, 32). However, the function of other viral proteins of PDCoV, especially the viral structure protein (S, E, M, N), has remained unclear. PDCoV N protein could change expression levels of many host immune proteins in the N-expressing PK-15 (PK-PDCoV-N) cells, such as HSP70 (33). However, it remains unclear whether PDCoV N protein is an antagonist in the porcine type I interferon signaling pathway and how to regulate the antiviral signaling pathway in porcine cells.

In this study, we confirmed and explained the mechanism by which PDCoV N protein antagonizes porcine IFN-β production. The results indicated that PDCoV N protein could directly target the porcine RIG-I and block its early activation by interfering its association with dsRNA and pRiplet- mediated K63-linked polyubiquitination.

## Materials and Methods

### Cell Culture and Virus

HEK293T cells (ATCC®CRL-3216™) and PK-15 cells (ATCC®CCL-33™) were obtained from the China Center for Type Culture Collection and maintained at 37°C in 5% CO_2_ in Dulbecco's Modified Ea-gle's medium (Gibco, USA) supplemented with 10% heat-inactivated fetal bovine se-rum (FBS) (Gibco, USA). The porcine jejunum intestinal cells (IPEC-J2) were obtained from the China Center for Type Culture Collection and maintained at 37°C in 5% CO_2_ in Roswell Park Memorial Institute (RPMI) 1,640 medium (Gibco, USA) with 10% heat-inactivated FBS (Gibco, USA). The recombinant VSV-GFP virus was generously provided by Dr. Sun Tao, Shanghai Jiao Tong University, China.

### Plasmids and Quantitative RT-PCR (qRT-PCR)

Total RNA was extracted by TRIZOL (Invitrogen). Then, 1 μg of RNA was used to synthesize cDNA using the ReverTra Ace qPCR RT reverse transcription (RT) master mix with genomic DNA (gDNA) remover (Toyobo, Osaka, Japan). The cDNA of PK-15 cells or PDCoV-positive sample were used as the templates to perform in 50 μL amplification reaction containing 2 μL of cDNA, 2 μL of forward and reverse primers (10 pmol), 10 μL 5 × PrimeSTAR GXLBuffer, 4 μL 2.5 nM dNTPs, 1 μL PrimeSTAR® GXL DNA Polymerase (TAKARA, Japan), and 29 μL ddH_2_O. The reaction procedure was 98°C for 3 min, followed by 36 cycles at 98°C for 15 s, 60°C for 30 s and 72°C for 1 min, and finally 72°C for 5 min. And then the full length or mutant coding sequence of porcine RLR signaling molecules were con-structed into the pcDNA3.1-Flag or pcDNA3.1-HA plasmid by ClonExpress®II One Step Cloning Kit (Vazyme, China), including porcine *RIG-I* (*pRIG-I*) (NM_213804.2), three *pRIG-I* mutants: the 2′CARD, a middle helicase domain (HEL), and the internal repressor domain (RD). porcine *MDA5* (*pMDA5*) (MF358967.1), porcine *MAVS* (*pMAVS*) (NM_001097429.1), porcine *TBK1* (*pTBK1*) (NM_001105292.1), porcine *TRAF3* (*pTRAF3*) (XM_021081629.1), porcine *IRF3* (*pIRF3*) (NM_213770.1), porcine *TRIM25* (*pTRIM25*) (XM_005656971.3), and porcine *Riplet* (*pRiplet*) (XM_003131735.4). The full-length coding sequence (CDS) of PDCoV-N was constructed into the pcDNA3.1-HA or pcDNA3.1-Myc plasmid vector. All the PCR primers are provided in Supplementary Table 1. The pGL3-pIFN-β-Luc and pGL3-pNF-κB-Luc plasmid were constructed according a previous study (34). HA-tagged ubiquitin (Ub) and Ub mutants (K48R, K63R, K48, K63) plasmids were kindly provided by Dr. Yuan Congli, Shanghai Jiao Tong University, China.

Quantitative RT-PCR (qRT-PCR) was performed using SYBR green Supermix (ABI-7500, Life, USA). The primer sequences are shown in Supplementary Table 1. The relative gene expression levels were calculated using the 2^−ΔΔCT^ method.

### Dual-Luciferase Reporter Gene Assay

PK-15 or IPEC-J2 cells were grown in 24-well plates. In selected experiments, the recombination or empty expression plasmids were cotransfected with the pGL3-pIFN-β-Luc and pRL-TK (an internal control for the normalization of the transfection efficiency) using Lipofectamine 2000 (InvivoGen, USA). After transfection for 24 h, poly(I:C) or VSV-GFP virus were transfected or infected for 16 h. The cells were then lysed, and the firefly luciferase and Renilla luciferase activities were measured using the Dual-Luciferase reporter assay system (Promega, USA). Data were shown as the relative firefly luciferase activities normalized to the Renilla luciferase activities from three independently conducted experiments.

### Western Blotting and Co-immunoprecipitation (Co-IP) Assay

HEK293T or PK-15 cells were transfected empty or recombinant plasmid. At 28 h post-transfection, the cells were harvested by adding lysis buffer (50 mM Tris-HCl (pH 7.4), 150 mM NaCl, 1% NP-40, 10% glycerin, 0.1% SDS, and 2 mM Na_2_EDTA) for 30 min at 4°C supplemented with a protease inhibitor cocktail, phenylmethylsulphonyl fluoride (PMSF), and a phosphatase inhibitor cocktail. The lysates were subjected to SDS-PAGE and electroblotted onto a polyvinylidene difluoride membrane (Bio-Rad, USA). The membranes were then analyzed for the expression proteins by immunoblotting using mouse Flag, Myc, β-actin, and rabbit HA antibodies, respectively. The β-actin or β-Tublin monoclonal antibody was used to detect the expression of β-actin or β-Tublin to confirm equal protein sample loading.

HEK293T or PK-15 cells in 10-cm culture dishes were cotransfected with the recombination expression plasmid or an empty vector for 28 h. The cells were lysed on ice for 20 min in 600 μl of lysis buffer (50 mM Tris-HCl (pH 7.4), 150 mM NaCl, 1% NP-40, 10% glycerin, 0.1% SDS, and 2 mM Na_2_EDTA) containing a protease inhibitor mixture plus the protease inhibitor PMSF. The cell lysates were then immunoprecipitated at 4°C with mouse anti-FLAG or anti-HA affinity gel (Biotool, USA) or mouse Myc monoclonal Ab with protein A+G agarose beads (Beyotime, China). The immunoprecipitants were washed four times with the protein lyse buffer and then subjected to western blotting analysis.

pRIG-I and dose-dependent PDCoV N expression plasmid or empty plasmid was cotransfected into the HEK293T cells for 28 h. Then, the dsRNA binding assay was implemented as described in a previous report (31).

### Indirect Immunofluorescence Assay (IFA)

PK-15 cells were seeded onto microscope coverslips, placed into 12-well plates, and allowed to reach approximately 80% confluence. At 24 h post transfection, the cells were fixed with 4% paraformaldehyde for 10 min and then permeabilized with methyl alcohol for 10 min at room temperature. After three washes with TBST, the cells were blocked with TBST containing 5% bovine serum albumin (BSA) for 1 h and then incubated separately with a mouse Flag monoclonal antibody against Flag-tagged protein (1:1000) or a rabbit HA polyclonal antibody against the HA-tagged PDCoV N protein (1:1000) for 1 h. The cells were then treated with Alexa Fluor 488-labeled anti-mouse secondary antibody or Alexa Fluor 555-labeled anti-rabbit secondary antibody for 1 h at room temperature and subsequently treated with 4′, 6-diamidino-2-phenylindole (DAPI) for 15 min at room temperature. The antibody and DAPI used in the present study were purchased from Beyotime in China. Fluorescent images were visualized and examined using a confocal laser scanning microscope (Fluoview ver. 3.1; Olympus, Japan).

### Ubiquitin Assay

To analyze the ubiquitination of porcine RIG-I, PK-15 or HEK-293T cells were cotransfected with Flag-RIG-I, HA-Ub, or HA-Ub mutants (K48R, K63R, K48, K63) and Myc-PDCoV-N for 28 h. The cells were washed twice in PBS supplemented with 10 mM NEM and lysed with 1% SDS lysis buffer (25 mM Tris-HCl, pH 7.4, 150 mM NaCl, 1% NP-40, 0.5% sodium deoxycholate, and 1% SDS) containing the protease inhibitor PMSF and 10 mM NEM. The mouse Flag-affinity gel was pretreated three times with 1 x TBS, and then the cell lysates were added and incubated for 3 h at 4°C. After washing them three times with 1xTBS, the immunoprecipitants were boiled at 100°C for 10 min and subjected to western blotting analysis.

### Statistical Analysis

Data are expressed as the mean ± standard deviation (SD) of three independent experiments. Student's *t*-tests were performed. Values of *p* < 0.05 were considered statistically significant and *p* < 0.01 were considered statistically highly significant.

## Results

### PDCoV N Protein Suppressed Poly(I:C) and VSV Induced IFN-β Production

**Figure 1:**
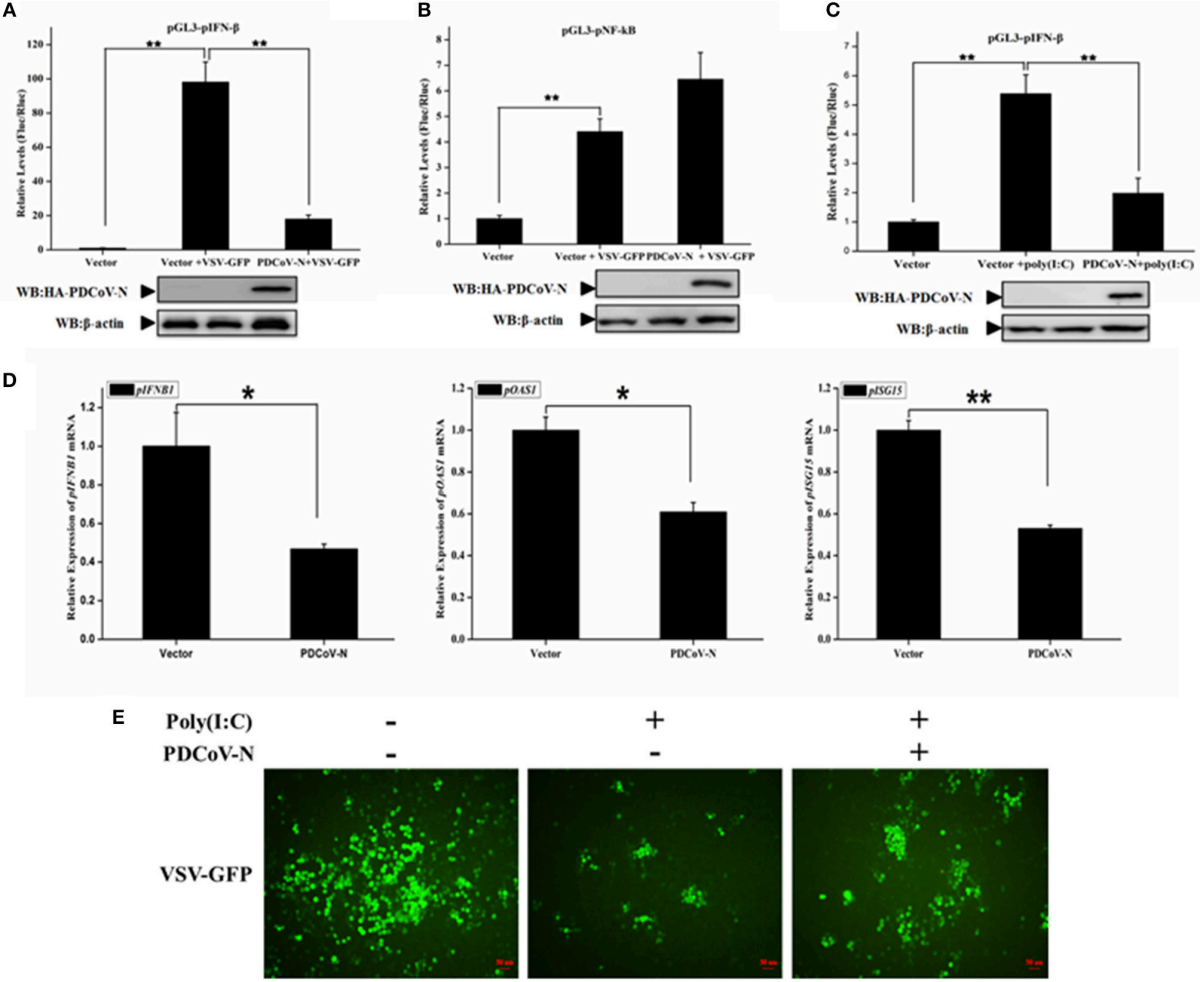
PDCoV N protein inhibited porcine IFN-β and ISGs expression. **(A–C)** PK-15 cells cultured in 24-well plates were transfected with porcine IFN-β-Luc (pGL3-pIFN-β) or porcine NF-κB-Luc (pGL3-pNF-κB) plasmid and pRL-TK plasmid with PDCoV N expression plasmid (pcDNA3.1-HA-PDCoV-N). At 18 h after transfection, cells were treated or untreated with VSV-GFP or Poly(I:C) (LMW). The cells were then subjected to dual-luciferase assays at 16 h post-treatment. The relative firefly luciferase activity was relative to that of an empty vector control. The *Renilla reniformis* luciferase activity was used to normalize. The expression of PDCoV N protein was detected by western blot with HA antibody. β-actin was detected as the loading control. **(D,E)** PK-15 cells cultured in 12-well plates were transfected with pcDNA3.1-HA-PDCoV-N or empty plasmid for 24 h, and then transfected with Poly(I:C) (LMW). **(D)** After 16 h post transfection, the cells were lysed by TRZOL to extract the total RNA. The qRT-PCR was used to detect the relative expression of porcine ISGs mRNA. **(E)** At 12 h post transfection, the VSV-GFP (MOI = 0.1) infected the PK-15 cells for another 16 h. The GFP was detected by fluorescence microscope. ^*^p < 0.05; ^**^p < 0.01.

To explore whether PDCoV N protein antagonizes the production of pIFN-β, PK-15 cells were cotransfected empty vector, or PDCoV-N expression plasmid (pcDNA3.1-HA-PDCoV-N) with the reporter plasmid (pGL3-pIFN-β) and pRL-TK (as internal control) for 18 h, and then infected with VSV-GFP (a recombinant VSV strain) or treated with poly(I:C) for 16 h. The cells were lysed for the dual-luciferase reporter assays. The results showed that the VSV-GFP or poly(I:C) induced porcine IFN-β-luc promoter activation was significantly suppressed by PDCoV-N protein in PK-15 cells (Figures 1A,C). However, the porcine NF-κB-Luc promoter activation was not inhibited by PDCoV-N protein in PK-15 cells, when infected with VSV-GFP virus (Figure 1B). To further prove that PDCoV N protein inhibits porcine IFN-β production, PK-15 cells were transfected with PDCoV-N expression or empty vector plasmid for 24 h and then transfected or non-transfected with poly(I:C) for 12 h. Total RNA was extracted from cells to detect the expression level of porcine IFN-β and several interferon-induced genes (ISGs) by real-time quantitative polymerase chain reaction (qRT-PCR). The results showed that PDCoV N overexpression could significantly suppress poly(I:C)-induced porcine *IFNB1* (*pIFNB1*), porcine *OAS1* (*pOAS1*), and porcine *ISG15* (*pISG15*) mRNA expression in PK-15 cells (Figure 1D). The results indicated that PDCoV N protein suppressed porcine IFN-β production. IFN-β could significantly inhibit the VSV replication in infected host cells. Hence, to further confirm the porcine IFN-β protein production decreased by PDCoV N protein, VSV-GFP viruses were used to infect PK-15 cells. The results showed that poly(I:C) could significantly suppress the VSV-GFP replication in PK-15 cells (Figure 1E). In accordance with the results of dual luciferase assays and qRT-PCR described above, the expression of PDCoV N protein could restore the proliferation of VSV-GFP virus in ploy(I:C)-treated PK-15 cells (Figure 1E). These data suggested that PDCoV N protein was an antagonist of porcine IFN-β.

### PDCoV N Protein Suppressed the Porcine RLR Signaling Pathway

**Figure 2:**
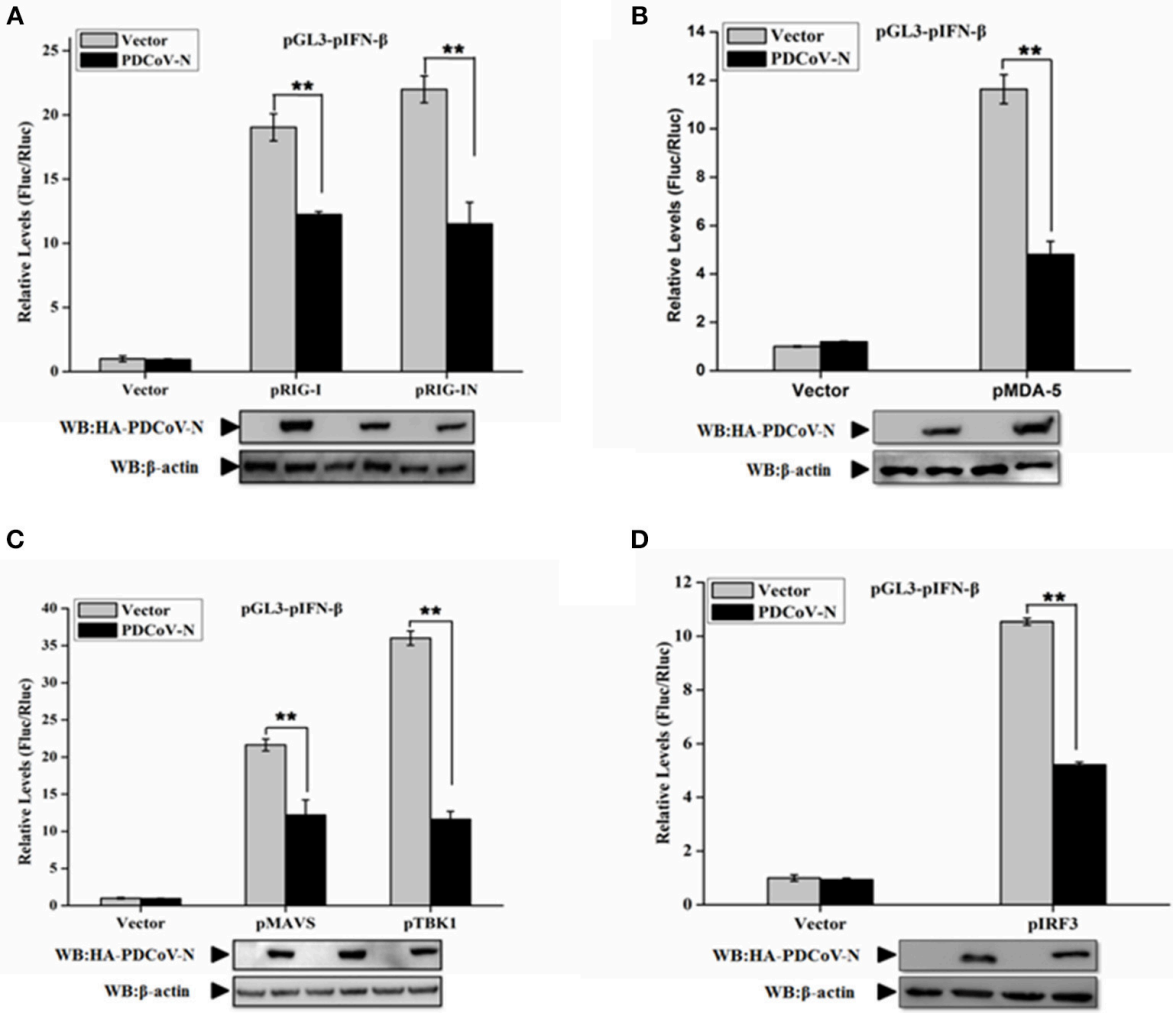
PDCoV N protein inhibited porcine IFN-β promoter activation by the porcine RLR signaling pathway. PK-15 cells were cotransfected with pGL3-pIFN-β,pRL-TL, and pcDNA3.1-HA-PDCoV-N along with constructed expression porcine RIG-I/RIG-IN (pRIG-I/pRIG-IN) **(A)**, porcine MDA5 (pMDA5) **(B)**, porcine MAVS (pMAVS) **(C)**, porcine TBK1 (pTBK1) **(C)**, or porcine IRF3 (pIRF3) **(D)**. Dual-luciferase assays were performed at 24 h post transfection. The relative firefly luciferase activity was relative to that of an empty vector control. The *Renilla reniformis* luciferase activity was used to normalize. Western blot was used to detect the protein expression of PDCoV N with HA antibody. The β-actin was as the loading control protein. All the experiments were independently performed three times. ^**^p < 0.01.

In the present study, we found PDCoV N protein was an antagonist of porcine IFN-β production (Figure 1). Therefore, to determine whether PDCoV N protein could block the porcine RLR-mediated type I IFN signaling pathway, we constructed several key porcine RLR (pRLR) signaling molecules from PK-15 cells, including pRIG-I, pRIG-IN (a pRIG-I mutant, only the 2′-CARD domain of pRIG-I (pRIG-IN)), pMDA5, pMAVS, pTBK1, and pIRF3. To investigate the function of PDCoV N protein in the porcine RLR pathway, PK-15 cells were co-transfected the key signaling molecules with the PDCoV N expression or empty vector plasmid, together with the pGL3-pIFN-β and pRL-TK. Compared with the empty vector, the overexpression of the porcine signaling molecules could clearly activate the pIFN-β promoter activation (Figure 2). However, the activation of the pIFN-β promoter induced by those signaling molecules was significantly inhibited by PDCoV N protein (Figure 2). The function of PDCoV N was confirmed by the consistent results in the IPEC-J2 cells (Supplementary Figure 1). These results indicated that the PDCoV N protein could suppress the porcine IFN-β by inhibiting the porcine RLR signaling pathway.

### PDCoV N Protein Interacted With Porcine RIG-I/TRAF3 Protein

**Figure 3:**
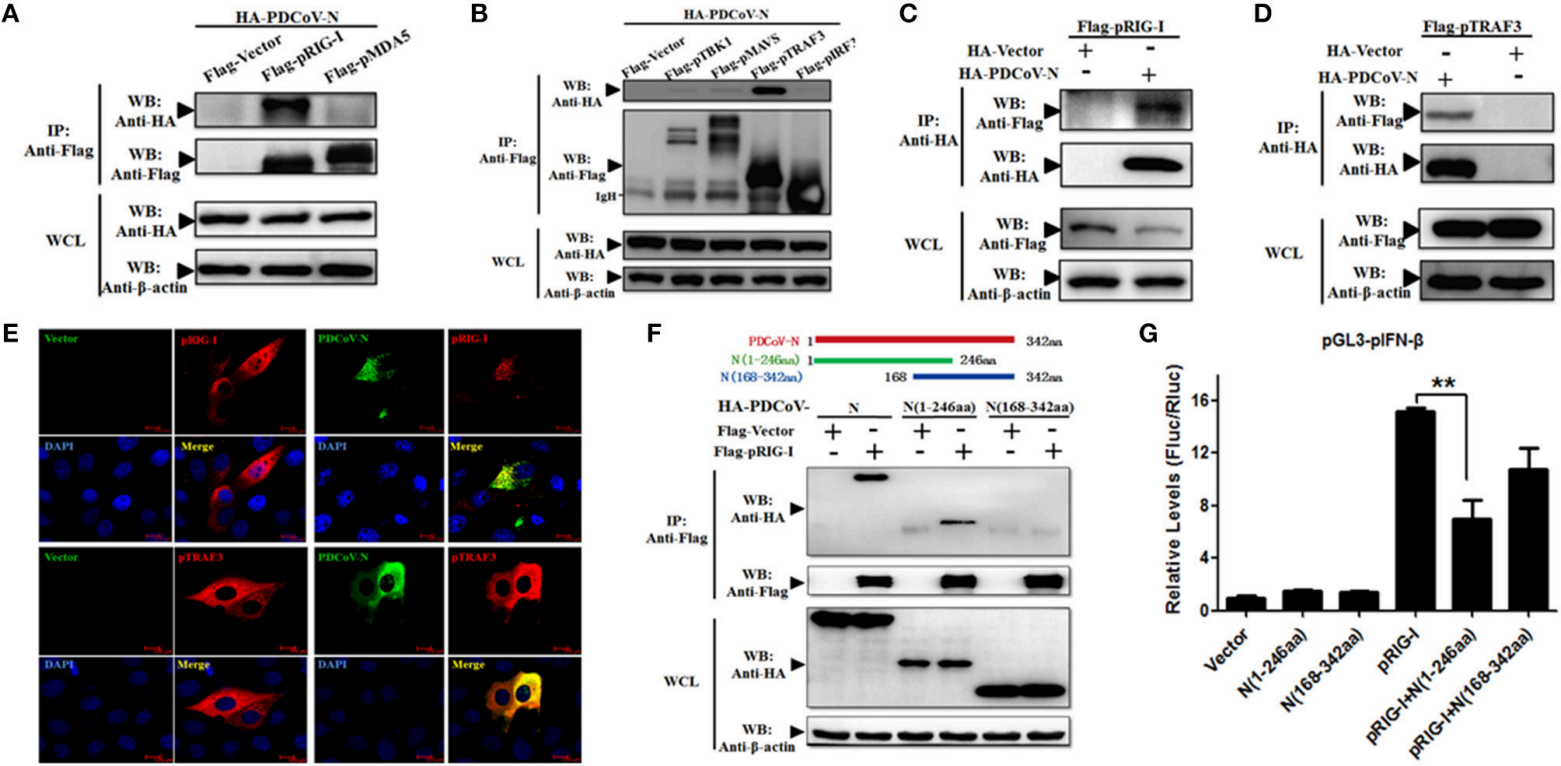
PDCoV N interacts with both pRIG-I and pTRAF3. HEK293T cells were co-transfected with HA-tagged PDCoV-N and Flag-tagged pRIG-I **(A–C)**, Flag-tagged pMDA5 **(A)**, Flag-tagged TBK1 **(B)**, Flag-tagged pMAVS **(B)**, Flag-tagged pTRAF3 **(B,D)** or Flag-tagged pIRF3 **(B)**, respectively. After 28 h of transfection, cells were lysed for co-immunoprecipitation (Co-IP) assay with anti-Flag (IP: Anti-Flag) or anti-HA (IP: Anti-HA) affinity gel. The WCLs and immunoprecipitants were detected by western blot with anti-Flag, anti-HA or anti-β-action antibody. **(E)** PK-15 cells were cotransfected with HA-tagged PDCoV-N and Flag-tagged empty plasmid as control or Flag-tagged pRIG-I, respectively. At 24 h after transfection, cells were fixed for IFA to detect the PDCoV-N (green) and pRIG-I (Red) with anti-HA and anti-Flag antibodies, respectively. 4′, 6-diamidino-2-phenylindole (DAPI, blue) stained the cell nuclei. Fluorescent images were acquired with a confocal laser scanning microscope (scar bar: 10 μm). **(F)** Schematic representation of PDCoV N protein fragments used for Co-IP analyses (up). HEK293T cells were cotransfected with Flag-tagged pRIG-I and HA-tagged full-length or truncated fragments of the PDCoV N protein. After 28 h of transfection, cells were lysed for Co-IP assay with anti-Flag affinity gel. The WCLs and immunoprecipitants were detected by western blot with anti-Flag, anti-HA, or anti-β-action antibody. **(G)** PK-15 cells were cotransfected with pGL3-pIFN-β, pRL-TL, and pcDNA3.1-Flag-pRIG-I alone or together with PDCoV N truncated fragments, respectively. Dual-luciferase assays were performed at 24 h post transfection. All the experiments were independently performed three times. ^**^p < 0.01.

To find the porcine RLR signaling molecular target of PDCoV N protein, the HEK293T cells were co-transfected with PDCoV N expression plasmid and several pRLR signaling molecules, pRIG-I, pMDA5, pMAVS, pTBK1, pTRAF3, or pIRF3, respectively. The whole cell lysates (WCLs) were immunoprecipitated with the anti-Flag affinity gel at 28 h post transfection. The results showed that pRIG-I and pTRAF3 were clearly coprecipitated with PDCoV N protein (Figures 3A,B). In addition, consistent results were presented, when the precipitation of pRIG-I or pTRAF3 with HA-PDCoV-N was analyzed by HA-affinity gel (Figures 3C,D). These results suggested that PDCoV N protein could directly interact with pRIG-I and pTRAF3. To further confirm the direct interaction between PDCoV N and pRIG-I in porcine cells, PK-15 cells were used to co-transfect the pRIG-I or pTRAF3 and PDCoV N expression plasmid. Indirect immunofluorescence assay (IFA) was performed at 24 h post transfection. The results showed that the PDCoV N protein and pRIG-I or pTRAF3 were co-localized in the cytoplasm in PK-15 cells (Figure 3E). All the data indicated that pRIG-I and pTRAF3 were the major targets of PDCoV N protein in the pRLR signaling pathway. RIG-I are the key cytoplasmic pathogen recognition receptors to recognize the RNA viruses. The activation of RIG-I was a prerequisite of IFN-β production by RLR signaling pathway. However, in the present study, PDCoV N protein could significantly block the pRIG-I-induced pIFN-β promoter activation (Figure 2A). Hence, pRIG-I was the main object in the following study. To investigate the interaction between PDCoV N protein and pRIG-I, two truncated mutants of PDCoV N protein(aa 1 to 246, and 168-342) were constructed and cotransfected with Flag-pRIG-I into HEK293T cells. The Co-IP results showed that the PDCoV N(1-246aa), but not another truncated region, could clearly coprecipitated with pRIG-I (Figure 3F). PDCoV N(1-246aa) was also significantly arrested pRIG-I induced porcine IFN-β promoter activation in PK-15 and IPEC-J2 cells (Figure 3G and Supplementary Figure 2). These results indicated that the N-terminal region was important for PDCoV N protein to interact with pRIG-I and interfere its function.

### PDCoV N Protein Suppressed Porcine RIG-I Activation

**Figure 4:**
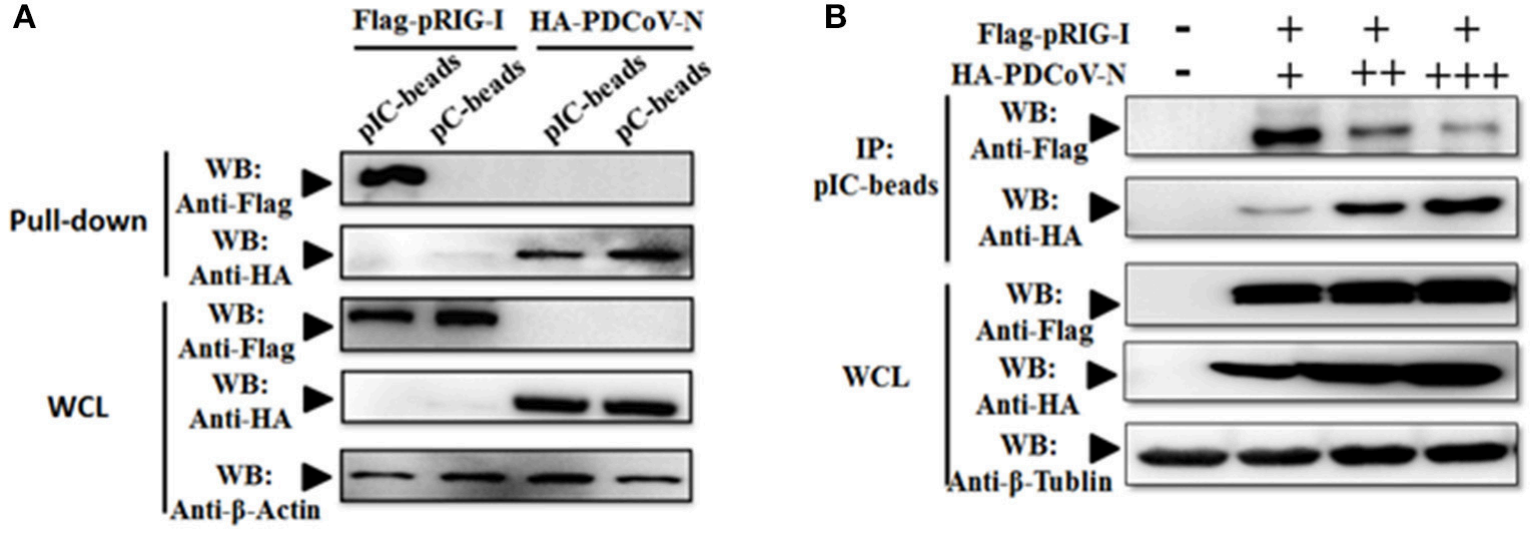
PDCoV-N suppressed the porcine RIG-I-binding dsRNA. **(A)** HEK293T cells were transfected with Flag-tagged pRIG-I or HA-tagged PDCoV expression plasmid. Cells were lysed for Co-IP by poly(I:C) (pIC) or poly(C)(pC)-beads at 28 h post-transfection. Western blot was used to detect the protein. **(B)** HEK293T cells were cotransfected with Flag-tagged pRIG-I with different dose HA-tagged PDCoV expression plasmid. After 28 h of transfection, the cells were lysed for immunoprecipitation by poly(I:C)-beads. Western blot was used to detect the immunoprecipitants and WCLs protein.

Previous researchers have reported that PDCoV NS6 inhibited IFN-β production by suppressing the double-stranded RNA (dsRNA) binding human RIG-I and MDA5 in HEK293T cells (31). In the present study, PDCoV N protein was proved as a directly pRIG-I binding protein (Figure 3). It is unclear whether PDCoV N could compete with porcine RIG-I to bind dsRNA. To verify this assumption, the poly(I:C) binding assay was performed. pRIG-I or PDCoV-N was eukaryotic expressed by transfection in HEK293T cells, respectively. After 28 h, the WCLs were immunoprecipitated with poly(I:C) (dsRNA) or poly(C) (ssRNA) beads. The results showed that PDCoV-N and pRIG-I could bind to the poly(I:C)-beads (Figure 4A). However, only PDCoV N protein could bind to the poly(C)-beads (Figure 4B). pRIG-I and dose-increased PDCoV-N expression plasmid were cotransfected in HEK293T cells. After 28 h, the WCLs were immunoprecipitated with poly(I:C)-beads. The results showed that the PDCoV N protein could decrease the binding of dsRNA and pRIG-I in a dose-dependent manner (Figure 3D). The results indicated that PDCoV N protein could compete the binding of dsRNA with pRIG-I.

**Figure 5:**
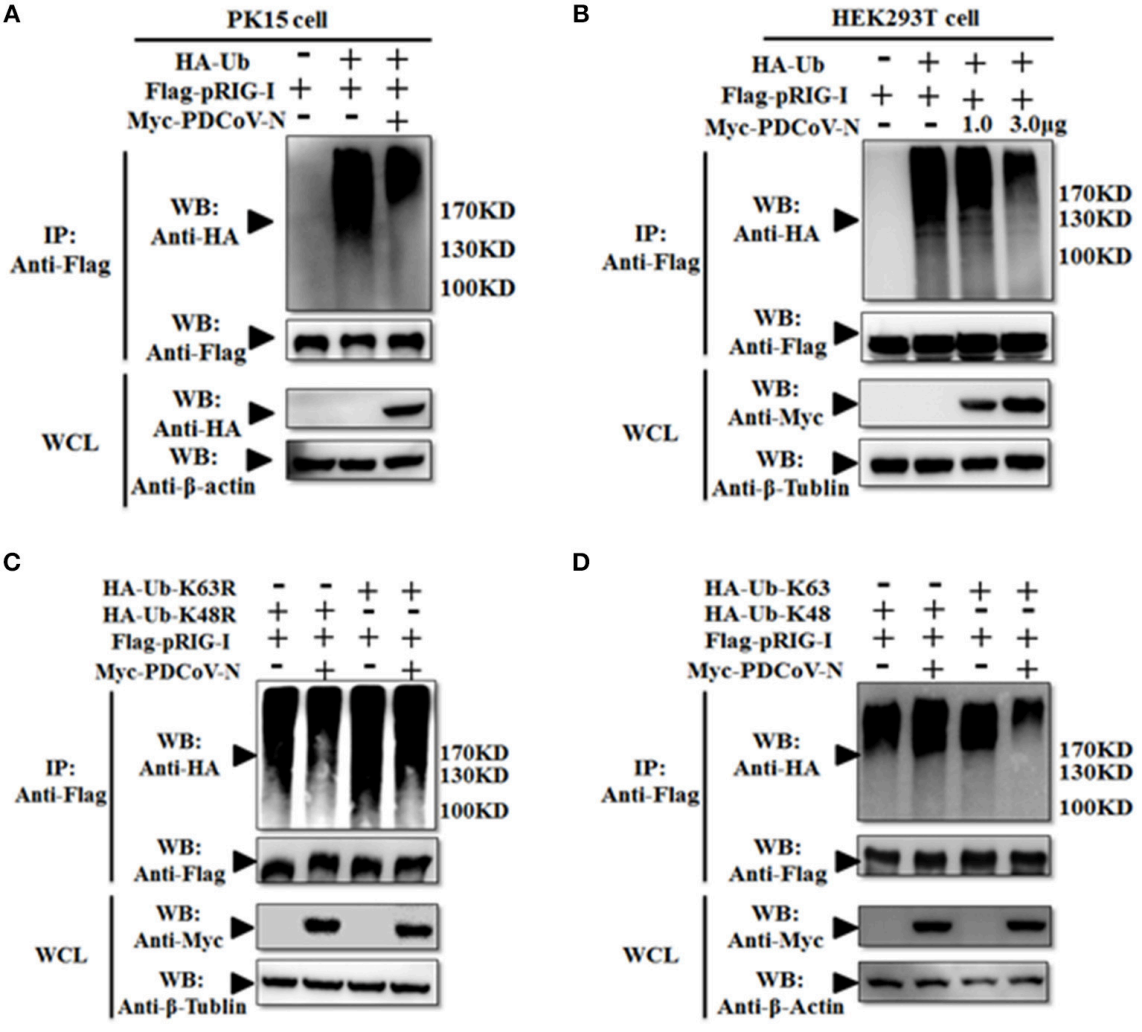
PDCoV N protein inhibited the pRIG-I K63-linked polyubiquitination. **(A,B)** HEK293T or PK-15 were cotransfected with Flag-tagged pRIG-I and Myc-tagged PDCoV N or empty control plasmid with HA-tagged ubiquitin (HA-Ub) or empty control plasmid. **(C,D)** HEK293T cells were cotransfected Flag-tagged pRIG-I and Myc-tagged PDCoV N or empty control plasmid with HA-Ub K48R, K63R, K48 only, or K63 only mutant plasmid or empty control plasmid, respectively. After 28 h of transfection, the cells were lysed for Co-IP by Flag-affinity gel. Western blot was used to detect the immunoprecipitants and WCLs protein.

However, in our previous study, we also found PDCoV N protein could directly interact with porcine RIG-I without dsRNA (Figures 3A,B). This indicated that PDCoV N protein may have other functions in regulated porcine RIG-I. Previous finding indicated that viral protein could also inhibit RIG-I activation by suppressing its polyubiquitination (21, 22, 25). To explore the role of PDCoV N protein in pRIG-I ubiquitination, pRIG-I expression plasmid were cotransfected with HA-tagged ubiquitin (Ub) and PDCoV N or empty plasmid in PK-15 or HEK293T cells for 28 h. The porcine RIG-I was purification by Flag affinity gel. Then, the polyubiquitination levels of pRIG-I were examined by western blot. The results showed that the polyubiquitination of pRIG-I was significantly increased, and the polyubiquitination were remarkably decreased by PDCoV N protein both in HA-tagged ubiquitin transfected PK-15 or HEK293T cells (Figures 5A,B). To further study which formation of pRIG-I linked-polyubiquitination was suppressed by PDCoV N protein, the ubiquitin K48R or K63R (HA-Ub-K48R or HA-Ub-K63R) mutant expression plasmid was used to co-transfect with porcine RIG-I together with PDCoV-N expression or empty plasmid in HEK293T cells. The results showed that PDCoV N protein could decrease the pRIG-I K48R but not K63R-induced polyubiquitination (Figure 5C). To further verify the results, the ubiquitin K63 or K48 (HA-Ub-K48 or HA-Ub-K63) only expression mutant plasmid was used to do the same experiments. PDCoV N protein could significantly decrease the pRIG-I K63-linked polyubiquitination, but not the K48-linked polyubiquitination (Figure 5D). All the results indicated that PDCoV N protein could suppress the pRIG-I polyubiquitination, especially the K63-linked polyubiquitination.

### PDCoV N Could Not Interact With Porcine Riplet and TRIM25

**Figure 6:**
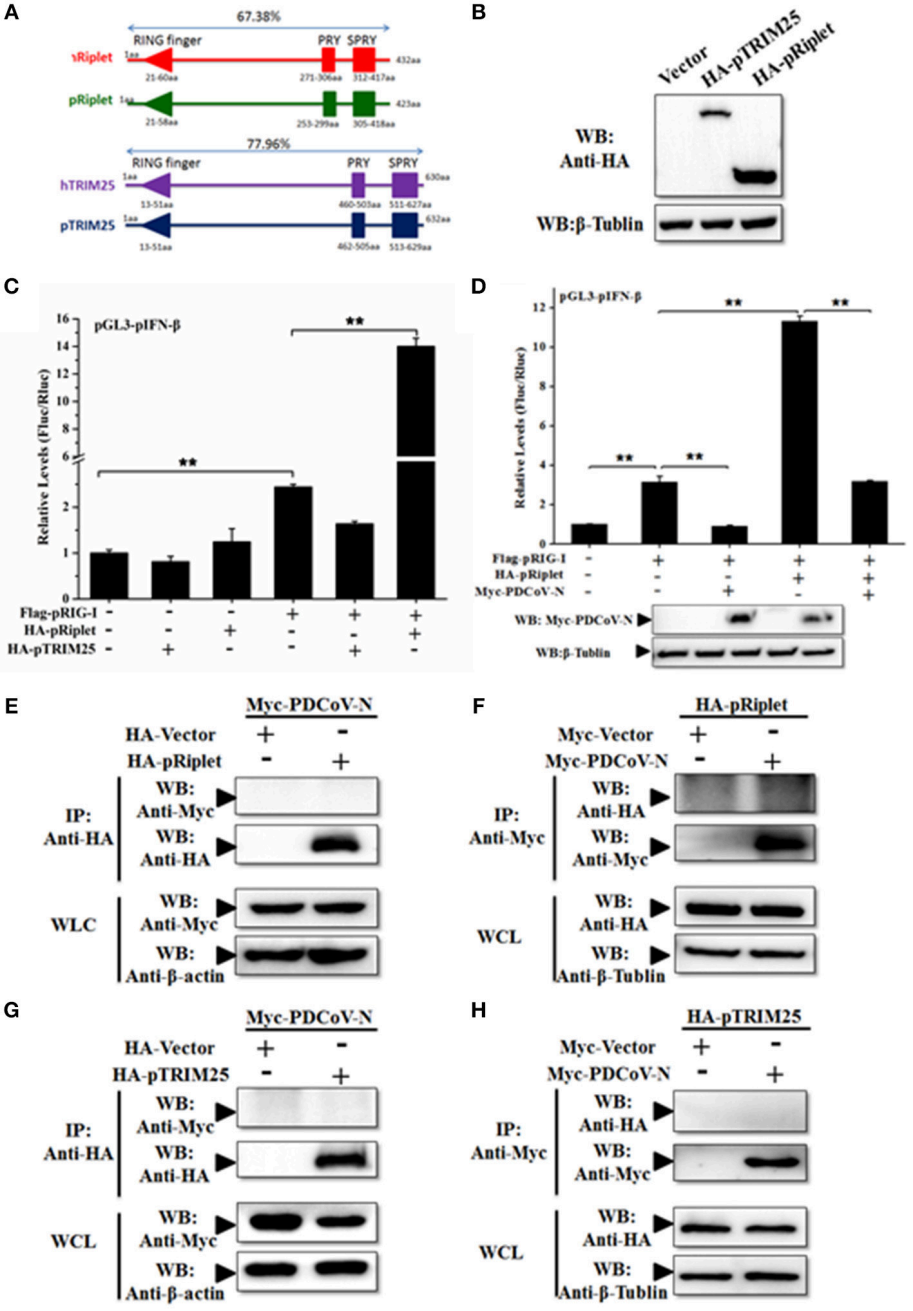
PDCoV N repressed porcine Riplet-induced pRIG-I activation. **(A)** The sequence alignment of porcine TRIM25 (pTRIM25) and Riplet (pRiplet) with human TRIM25 (hTRIM25) and Riplet (hRiplet), respectively, by Clustal Omega (https://www.ebi.ac.uk/Tools/msa/clustalo/). **(B)** HEK293T cells were transfected with the HA-tagged pTRIM25 or HA-tagged pRiplet expression plasmid. After 28 h of transfected, the WCLs were detected with anti-HA antibody by western blot. **(C)** PK-15 cells were co-transfected with pRIG-I, pGL3-pIFN-β, and pRL-TK plasmid with pTRIM25 or pRiplet, the empty plasmid as the control. Dual-luciferase assays were performed at 24 h post transfection. **(D)** PK-15 cells were co-transfected with pRIG-I, pGL3-pIFN-β, and pRL-TK plasmid with pRiplet and Myc-PDCoV-N, the empty plasmid as the control. Dual-luciferase assays were performed at 24 h post transfection. All the experiments were independently performed three times. **(E,G)** HEK293T cells were cotransfected with HA-tagged pRiplet or HA-tagged pTRIM25 or HA-empty plasmid as the control with Myc-tagged PDCoV-N expression plasmid, respectively. **(F,H)** HEK293T cells were co-transfected with Myc-tagged PDCoV (Myc-PDCoV-N) or Myc-empty plasmid as the control expression plasmid with HA-tagged pRiplet **(F)** or HA-tagged pTRIM25 **(H)** plasmid, respectively. After 28 h of transfection, the cells were lysed for Co-IP with anti-Myc monoclonal antibody (IP: Anti-Myc). Western blot was used to detect the immunoprecipitants and WCLs protein. ^**^p < 0.01.

TRIM25 and Riplet protein were the two important regulated K63-polyubiquitinations of human RIG-I activation. To further explore the mechanism of PDCoV-N suppression of pRIG-I K63-polyubiquitination, porcine TRIM25 (pTRIM25) and Riplet (pRiplet) were mainly analyzed. pTRIM25 and pRiplet have only 77.93 and 67.38% identity to human TRIM25 (hTRIM25) and Riplet (hRiplet) by amino acids sequence analysis, respectively (Figure 6A). The pTRIM25 or pRiplet expression plasmid was constructed and expressed in PK-15 cells (Figure 6B). To explore the function of pTRIM25 or pRiplet in pRIG-I induced IFN-β production, the pTRIM25, or pRiplet was cotransfected with pRIG-I and pGL3-pIFN-β-Luc and pRL-TK-Luc plasmid in PK-15 cells for 28 h. The results showed that pRiplet could significantly promote pRIG-I-induced pIFN-β-Luc activation (Figure 6C). However, pTRIM25 could not influence pRIG-I-induced pIFN-β-Luc activation (Figure 6C). The consistent results were observed in the IPEC-J2 cells (Supplementary Figure 3A). The results showed that pRiplet was the more important protein in activating pRIG-I. To determine whether PDCoV N protein could influence the pRiplet-induced pRIG-I-induced pIFN-β production, pRIG-I and pRiplet were co-transfected with pGL3-pIFN-β-Luc, pRL-TK-Luc, and PDCoV N or empty plasmid for 28 h in PK-15 cells. The PDCoV N protein was detected in the WCLs by western blot (Figure 6D). The results showed that PDCoV N protein could prominently inhibit pIFN-β-Luc activation which is induced by pRiplet-promoted pRIG-I (Figure 6D). The consistent results were confirmed in the IPEC-J2 cells (Supplementary Figure 3B).

TRIM25 and Riplet were also the targets of a virus to suppress the type I IFN signaling pathway (21, 25). The interactions between PDCoV N protein and pTRIM25 or pRiplet are explored in the present study. The PDCoV N and pTRIM25 or pRiplet expression plasmid were co-transfected in the HEK293T cells for Co-IP analysis. The results showed that PDCoV N protein was neither coprecipitated with pTRIM25 nor pRiplet by HA-tagged affinity gel (Figures 6E,G). The pTRIM25 or pRiplet was also undetectable in anti-Myc immunoprecipitation (Figures 6F,H). The results demonstrated that PDCoV N protein could not directly interact with pTRIM25 or pRiplet.

### PDCoV N Protein Interfered pRiplet Induced pRIG-I K63-Linked Polyubiquitination

**Figure 7:**
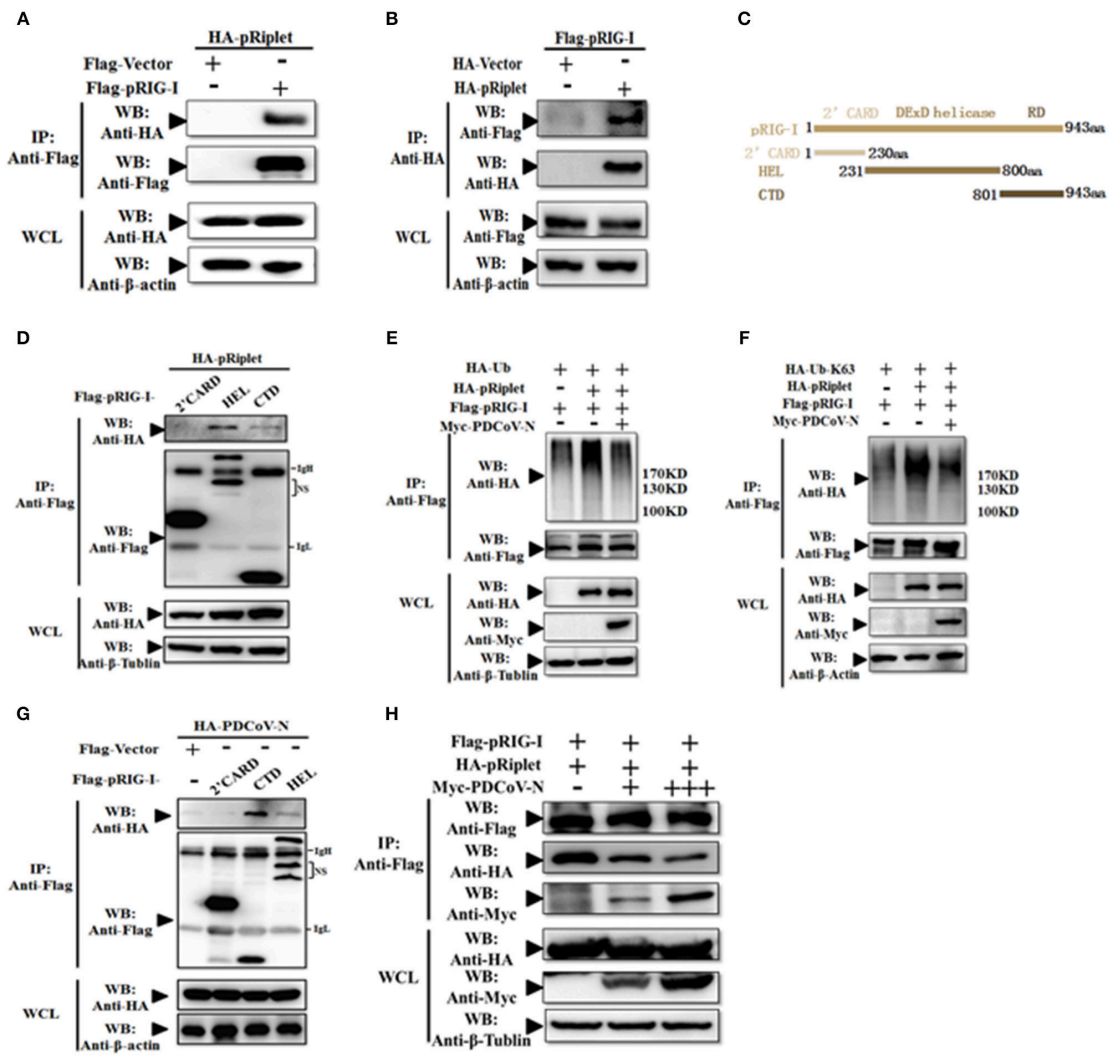
PDCoV N protein inhibited pRiplet-induced pRIG-I K63-polyubiquitination. **(A,B)** HEK293T cells were co-transfected with HA-tagged pRiplet or Flag-tagged pRIG-I or empty plasmid as the control with Flag-tagged pRIG-I or HA-tagged PDCoV-N expression plasmid, respectively. After 28 h of transfection, the cells were lysed for Co-IP by Flag-affinity gel or HA-affinity gel. **(C)** Schematic representation of porcine RIG-I fragments used for Co-IP analyses. **(D,G)** HEK293T cells were co-transfected with HA-tagged pRiplet **(D)** or HA-tagged PDCoV-N **(G)** with three porcine RIG-I fragments (Flag-pRIG-I-2′CARD, Flag-pRIG-I-HEL, Flag-pRIG-I-CTD), or expression plasmid, respectively. After 28 h of transfection, the cells were lysed for Co-IP by Flag-affinity gel. **(E,F)** HEK293T cells were co-transfected with Flag-tagged pRIG-I and HA-Ub or HA-Ub-K63 with Myc-tagged PDCoV-N or HA-tagged pRiplet or empty control plasmid, respectively. After 28 h of transfection, the cells were lysed for Co-IP by Flag-affinity gel. **(H)** HEK293T cells were co-transfected with HA-tagged pRiplet and Flag-tagged pRIG-I with Myc-tagged PDCoV-N or empty plasmid as the control expression plasmid, respectively. After 28 h of transfection, the cells were lysed for Co-IP by Flag-affinity gel. Western blot was used to detect the immunoprecipitants and WCLs protein.

To explore the mechanism of PDCoV N protein-repressed porcine Riplet-induced pRIG-I activation, first, pRiplet and pRIG-I plasmid were cotransfected in HEK293T cells for 28 h to analyze their relationship by Co-IP assay. The results showed that pRiplet could directly interact with pRIG-I (Figures 7A,B). Then, three pRIG-I mutants expression plasmids (only contain the 2′CARD, HEL, or RD domain) or empty plasmid was co-transfected with pRiplet expression plasmid in HEK293T cells (Figure 7C). The Co-IP results showed that pRiplet bound to the HEL and RD domain but not the 2′CARD domain (Figure 7D). Further analysis found that pRIG-I ubiquitination or K63-linked polyubiquitination was remarkably increased when the cells were cotransfected with pRiplet (Figures 7D,E). However, the PDCoV N protein expression could significantly decrease the pRiplet-induced pRIG-I ubiquitination and K63-linked polyubiquitination (Figures 7C,D).

To further understand the mechanism of PDCoV N protein-binding pRIG-I, three pRIG-I mutants expression plasmids or empty plasmid was co-transfected with PDCoV N expression plasmid in HEK293T cells for 28 h. The Co-IP results showed that PDCoV N protein could bind to both the HEL and RD domain but not the 2′CARD domain (Figure 7F). The binding domains were consistent with pRiplet (Figure 7D). This suggested that PDCoV N might interfere the binding between pRIG-I and pRiplet. To confirm the assumption, the pRiplet and pRIG-I were cotransfected with increasing doses of PDCoV N expression plasmid in HEK293T cells. The Co-IP results showed that with the PDCoV N protein increasing, the pRiplet binding pRIG-I was significantly decreased in the coprecipitates (Figure 7H). All above results proved that PDCoV N protein could interfere with pRiplet binding pRIG-I to inhibit the pRiplet-mediated pRIG-I K63-polyubiquitination.

## Discussion

Innate immunity is the first line to defend against virus infections, especially the production of IFNs and ISGs. Viruses also have diverse means to evade the host innate immune response. PDCoV infection suppressing IFN-β production has also been reported (30). Research has indicated that PDCoV non-structure protein 5 (Nsp5) encoded a 3C-like protease, which has lyase activity, to interrupt the IFN-β signaling pathway by decomposing human NEMO in HEK293T cells (32). PDCoV Nsp5 also could cleavage the signal transducer and activator of human transcription 2 (STAT2), an essential component of transcription factor complex ISGF3, to antagonize IFN-β signaling in HEK293T cells (35). PDCoV accessory protein 6 (NS6) was proved as another IFN-β antagonist by interfering with the binding of human RIG-I/MDA5 to double-stranded RNA in HEK293T cells (31). In the present study, we found that PDCoV N is another antagonist of porcine IFN-β production induced by poly(I:C), VSV, and porcine RLR-signaling molecules (Figures 1, 2 and Supplementary Figure 1).

Although TGEV N protein has no effect on IFN-β production, coronavirus N protein as an antagonist of the RLR-mediated IFN-β signaling pathway has been confirmed in PEDV, SARS-CoV, MHV, and MERS-CoV (25, 26, 36, 37). However, previous reports have indicated that the different coronavirus N protein as an IFN antagonist intervened with the RLR signaling pathway by targeting different signaling proteins. SARS-CoV N protein antagonized IFN-β in the initial signaling pathway (38). The protein activator of protein kinase R (PACT) is a cellular dsRNA-binding protein potentiating IFN production by binding to RIG-I and MDA5 (39, 40). The mouse hepatitis virus (MHV) and SARS-CoV N protein could interact with PACT to suppress the dsRNA translocated to RIG-I and MDA5 (36). The PEDV N protein was found to interact with human TBK1 and IKKε to inhibit the human TBK1/IRF3 complex confirmed to suppress IFN-β production (26). In the present study, we proved that PDCoV N directly interacts with pRIG-I and pTRAF3 (Figure 3), which is different from the previously reported coronavirus N protein, such as PEDV, SARS-CoV, MERS-CoV, and MHV.C-terminal region of SARS-CoV nucleocapsid protein was critical for antagonizing IFN-β response (38). However, the N-terminal region was necessary for PDCoV N protein to inhibit porcine IFN-β response by targeting pRIG-I (Figures 3F,G). Furthermore, we also found that PDCoV N protein significantly inhibited the porcine IRF3 induced IFN-β promoter activation. However, the PDCoV N protein was showed not interacting with porcine IRF3 protein, suggesting another target signal molecule of PDCoV N protein may exist in the downstream of porcine IRF3.

RIG-I and MDA5 contain three similar structures: 2′CARDs, HEL, and C-terminal domain (CTD) (15). RIG-I and MDA5 sense distinct RNAs depending on their different C-terminal structures. The C-terminal structure of human RIG-I contains a conservative internal repressor domain (RD), but this is not found in MDA5 (15). In the present study, we found that PDCoV N protein could directly interact with porcine RIG-I but not porcine MDA5. In addition, the RD domain was important for PDCoV N protein interacting with porcine RIG-I (Figure 6F). This may explain why PDCoV N protein specifically binds to porcine RIG-I.

**Figure 8:**
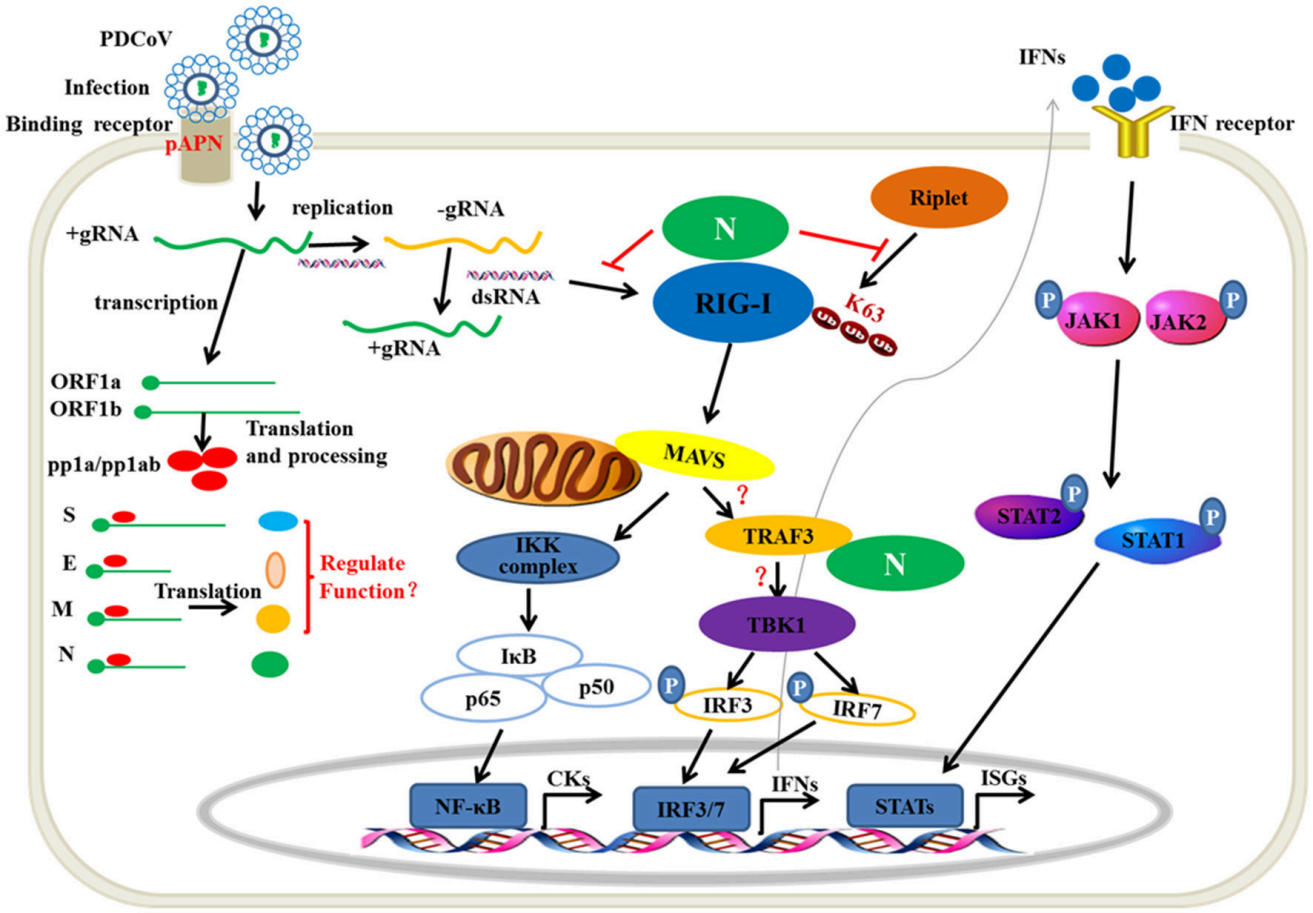
The mechanism of PDCoV N protein interfered with the type I IFN response. PDCoV infect the host cells by attaching to the celluar receptor porcine APN. In the cytoplasm, PDCoV release the viral genomic RNA and then complete the transcription and replication process. The ssRNA as well as dsRNA as replicative intermediate are sensed by porcine innate nucleic acids sensors in the cytoplasm to induce the IFNs production, such as porcine RIG-I. The E3 ubiquitin ligase Riplet is shown to active RIG-I by K63-linked polyubiqutination. Activation of JAK-STAT pathway by IFNs binding its receptor induces the production of ISGs, such as *OAS1, ISG15*. In the present, PDCoV N protein can block the porcine RIG-I dsRNA-binding and interferes with porcine Riplet-mediated porcine RIG-I K63-linked polyubiquitination to restrain the type I IFN response. PDCoV N protein also interacts with porcine TRAF3. However, the mechanism of the association is unclear. The other viral structure proteins are unclear in regulating type I IFN response.

Viral nucleic acids as pathogen associated molecular pattern are sensed by the host pattern recognition receptors after a viral infection. RIG-I and MDA5 are the major members of intracytoplasmic pathogenic molecular pattern recognition receptors that sense the intracellular viral RNA. Hence, one mechanism of the RNA virus escaping from host immune surveillance is to protect its ssRNA or dsRNA not to be recognized by RIG-I and MDA5. The MERS 4a protein is a dsRNA-binding protein that interacts with PACT in an RNA-dependent manner but not with RIG-I or MDA5, which suppresses the PACT-induced activation of RIG-I and MDA5 (41). PDCoV accessory protein 6 (NS6) could not bind dsRNA or ssRNA. However, eukaryotic-expression of PDCoV NS6 in HEK293T cells has been found to directly interact with human RIG-I and MDA5, and suppresses the host recognition of dsRNA (31). In the present study, PDCoV N protein was proven as a new member competing with porcine RIG-I binding dsRNA (Figure 4B). This is a mechanism of PDCoV N protein suppressing porcine IFN-β induction (Figure 8). It is noteworthy that the NS6 protein must be synthesized in infection cells, unlike the N protein, which can come from the parent virus itself. Therefore, we propose a hypothesis that there may be a synergistic effect between PDCoV N protein and NS6 protein. In the early stage of virus infection, especially before the synthesis of NS6 protein, the competitive binding dsRNA of PDCoV N protein inhibits the activation of pRIG-I. With the synthesis and expression of NS6 protein, the function of PDCoV N protein is replaced by NS6, which is conducive to N protein binding the viral gRNA to complete the assembly of progeny virus.

In addition, PDCoV N protein could inhibit porcine RIG-I or MDA5-induced pIFN-β promoter activation. The results indicated that PDCoV N protein may have another function to regulate the porcine RIG-I activation or suppress the downstream signaling transduction. TRAFs have an important role in signal transduction to regulate the immune and inflammatory responses. The C-terminal domain of TRAFs mediates its oligomerization and the association with upstream or downstream effector protein. TRAF3 has been considered the important signaling molecule as the bridge between MAVS and TBK1 (19). SARS-CoV papain-like protease could negatively regulate IRF3 activation by interacting with the STING-TRAF3-TBK1 complex (42). SARS-CoV M protein could suppress the TBK1/IKKε-dependent activation of IRF3/IRF7 by preventing the formation of TRAF3/TANK/TBK1/IKKε complex (43). The C-terminal effector domain of AIV NS1 also interacts with the TRAF3 protein to decrease its K63-linked polyubiquitination and disrupt the formation of the MAVS/TRAF3 complex (44). In the present study, we confirmed that PDCoV N protein also directly interacted with porcine TRAF3 (Figure 3). The mechanism of PDCoV N protein targeting porcine TRAF3 to mediate the type I interferon signaling pathway is unclear and is currently being explored by our team.

Ubiquitination is an important posttranslational modification in regulating the activation of signaling molecules. The E3 ubiquitin ligases TRIM25, TRIM4, and Riplet were proven to catalyze the K63-linked polyubiquitination of RIG-I in CARDs and CTD, respectively (17, 45–47). Suppressing the ubiquitin ligases binding with its target protein is another means of virus interfering with the host IFN-β production. AIV NS1 protein also could suppress RIG-I activation by species-specific interaction with TRIM25 or Riplet (21). The West Nile virus NS1 also antagonizes IFN-β production by inhibiting RIG-I and MDA5 K63-linked polyubiquitination (22). The SARS-CoV N protein directly interacts with human TRIM25 to suppress the RIG-I K63-linked polyubiquitination (25). The MERS-CoV N protein has a similar function as SARS-CoV N to inhibit RIG-I activation (25). However, we found that PDCoV N protein neither interacts with pTRIM25 nor pRiplet (Figures 5D–G). In the present study, we also found that pTRIM25 could not promote the activation of pRIG-I induced IFN-β promoter (Figure 6C). It indicated that the role of homologous proteins among different species might be different in regulating the activation of RLR signaling pathway. This may be an important reason for coronavirus N protein interaction with different proteins to regulate the host innate immunity in different hosts.

The mechanism of Riplet-mediated RIG-I activation remains unclear. hRiplet could promote the K63-linked polyubiquitination of the RIG-I CARD domain, which was indispensable for interaction between Riplet and RIG-I (34, 48). However, hRiplet was also found mainly interacting with the RIG-I RD domain and promoting the RD domain K63-linked polyubiquitination to release RIG-I autorepression (16, 47). In the present study, we found that pRiplet, as the homologous gene of hRiplet, could directly interact with pRIG-I, which was dispensable for the HEL and RD domain but not the CARD domain (Figure 7). pRilet mediated pRIG-I activation by increasing the pRIG-I K63-linked polyubiquitination. However, PDCoV N protein could suppress the pRiplet-mediated pRIG-I activation to induce IFN-β production. Further exploration demonstrated that PDCoV N protein blocked the pRIG-I K63-linked polyubiquitination by interfering pRiplet binding to pRIG-I (Figure 8). Two theories of the interfering binding mechanism might exist: First, because of the same target domains of PDCoV N protein and pRiplet binding pRIG-I, they may be competitively binding pRIG-I. On the other hand, we found that pRiplet mainly binds to the HEL domain of pRIG-I (Figure 7D), while PDCoV N protein mainly binds to the CTD of pRIG-I (Figure 7G). The human RIG-I CTD could interact with the HEL and CARD domain to self-repression (15). Hence, another reason is that the protein conformation of pRIG-I might be changed after binding to PDCoV N protein, which leading the pRiplet could not interact with pRIG-I. To our knowledge, this is the first time that coronavirus N protein has been reported to interfere the host immune system by directly targeting host RIG-I. These results provide insight into a novel mechanism of PDCoV inhibiting the host antiviral response.

## Author Contributions

YY, SJ, and JL conceived and designed the experiments. JL, LS, and ZW performed the experiments and analyzed data. JL, MJ, and WH wrote the manuscript. All authors reviewed, revised, and approved the final manuscript.

### Conflict of Interest Statement

The authors declare that the research was conducted in the absence of any commercial or financial relationships that could be construed as a potential conflict of interest.
