# Supplementary material for: Porcine Deltacoronavirus Nucleocapsid Protein Suppressed IFN-β Production by Interfering Porcine RIG-I dsRNA-Binding and K63-Linked Polyubiquitination
Source: Front Immunol. 2019 May 9;10:1024. doi: 10.3389/fimmu.2019.01024 (PMC6521028; doi:10.3389/fimmu.2019.01024)
Supplement: Supplementary file 1 [file Table_1.DOCX]

Supplementary Material

Porcine deltacoronavirus nucleocapsid protein suppressed IFN-β production by interfering porcine RIG-I dsRNA-binding and K63-linked polyubiquitination.

Ji Likai^1^, Li Shasha^1^, Zhu Wenxian^1^, Ma Jingjiao^1^, Sun Jianhe^1^, Wang Hengan^1^, Yan Yaxian^1*^

*** Correspondence:** Dr. Yan Yaxian, School of Agriculture and Biology, Shanghai Jiao Tong University, Shanghai Key Laboratory of Veterinary Biotechnology, Shanghai, People's Republic of China. yanyaxian@sjtu.edu.cn

# Supplementary Table 1.

# ****Table 1. The PCR and qRT-PCR primers****

| **Primer name** | **Sequence of oligonucleotide (5’-3’)** | **Purpose** |
| --- | --- | --- |
| *pRIG-I* | F: CTAGCGTTTAAACTTAAGCTTATGACAGCAGAGCAGCGGC | Full length  cloning |
|  | R: GTCCTTGTAATCCATGCGGCCGCACTCAAGGTTGCCCATTCCC |  |
| *pRIG-I-*2’CARD  (pRIG-IN) | F: CTAGCGTTTAAACTTAAGCTTATGACAGCAGAGCAGCGGC | Truncated  cloning |
|  | R: GTCCTTGTAATCCATGCGGCCGCAAGAGGAACACGAATTCTGACT |  |
| *pRIG-I* -HEL | F: CTAGCGTTTAAACTTAAGCTTATGTCAGAAGCACCTCATACTTAC | Truncated  cloning |
|  | R: GTCCTTGTAATCCATGCGGCCGCGTTTTCCTTGATTATCCCTGA |  |
| *pRIG-I* -CTD | F: CTAGCGTTTAAACTTAAGCTTATGCCAGAACCTGTGCCTGATAAG | Truncated  cloning |
|  | R: GTCCTTGTAATCCATGCGGCCGCACTCAAGGTTGCCCATTCCC |  |
| *pMDA5* | F: CTAGCGTTTAAACTTAAGCTTATGTCGTCGGATGGGTATTCC | Full length  cloning |
|  | R: GTCCTTGTAATCCATGCGGCCGCAGTCCTCATCACTAGACAAACAATATTCT |  |
| *pMAVS* | F: CTAGCGTTTAAACTTAAGCTTATGACGTTTGCCGAGGACAA | Full length  cloning |
|  | R: GTCCTTGTAATCCATGCGGCCGCACTGGGGCAGGCGCCGCC |  |
| *pTRAF3* | F: CTAGCGTTTAAACTTAAGCTTATGACACACAGAATGGAGCCG | Full length  cloning |
|  | R: GTCCTTGTAATCCATGCGGCCGCAGGGGTCAGGCAGATCCG |  |
| *pTBK1* | F: CTAGCGTTTAAACTTAAGCTTATGCAGAGCACTTCTAATCATCTTTG | Full length  cloning |
|  | R: GTCCTTGTAATCCATGCGGCCGCAAAGACAGTCAACATTGCGAAGG |  |
| *pIRF3* | F: CTAGCGTTTAAACTTAAGCTTATGGGAACTCAGAAGCCTCGG | Full length  cloning |
|  | R: GTCCTTGTAATCCATGCGGCCGCAGAAATCCATGTCCTCCACCAG |  |
| PDCoV-N  N(1-246aa)  N(168-342aa) | F: TAAGCTTATGGCCGCACCAGTAGTCCCT | Full length  and truncated cloning |
|  | R: CGGTACCCACGCTGCTGATTCCTGCT  R: CGGTACCGGCCAGCGAAAAGCATTTCC  F: TAAGCTTCAGCCCAGGAAACGCGACC |  |
| *pRiplet* | F: CCAGATTACGCTTCGGGTACCATGGCGGGCCCGGACGCCG | Full length  cloning |
|  | R: TGCTGGATATCTGCAGAATTCTTATAGGTTTACCTGCCTTACAGTCAG |  |
| *pTRIM25* | F: CCAGATTACGCTTCGGGTACCATGGCGGAACTGTGCCCCCTG | Full length  cloning |
|  | R: TGCTGGATATCTGCAGAATTCCTACCTGGTGGAGCAGATGGAG |  |
| *pIFNB1* | F: CATCCTCCAAATCGCTCTC | qRT-PCR |
|  | R: TCATCCTATCTTCGAGGCAA |  |
| *pISG15* | F: GCAGCAACGCCTATGAGGTCT | qRT-PCR |
|  | R: AGGCTTGAGGTCATACTCCCC |  |
| *pOAS1* | F: CTGAGGAACCGACCAACC | qRT-PCR |
|  | R: TTTCCTGCTTCCTTGCTCC |  |
| *pGAPDH* | F: TTTAACTCTGGCAAAGTGGACA | qRT-PCR |
|  | R: GGCCTTTCCATTGATGACAAGC |  |

**Not: *p*:porcine; F: forward primer; R:** **reverse primer.**

**Supplementary** **Figure 1**. **PDCoV N protein inhibited porcine IFN-β promoter activation by the porcine RLR signaling pathway.** IPEC-J2 cells were cotransfected with pGL3-pIFN-β，pRL-TL, and pcDNA3.1-HA-PDCoV-N along with constructed expression porcine RIG-I/RIG-IN(pRIG-I/pRIG-IN) **(A)**, porcine MDA5 (pMDA5), porcine MAVS (pMAVS)，porcine TBK1 (pTBK1) or porcine IRF3 (pIRF3) plasmids. Dual-luciferase assays were performed at 24 h post transfection. The relative firefly luciferase activity was relative to that of an empty vector control. The Renilla reniformis luciferase activity was used to normalize. All the experiments were independently performed three times. *p<0.05; **p < 0.01.

**Supplementary** **Figure 2. PDCoV N protein inhibited pRIG-I induced porcine IFN-β promoter activation.** IPEC-J2 cells were cotransfected with pGL3-pIFN-β，pRL-TL, and pRIG-I expression plasmid alone or along with PDCoV N truncated protein expression plasmids. Dual-luciferase assays were performed at 24 h post transfection. The relative firefly luciferase activity was relative to that of an empty vector control. The Renilla reniformis luciferase activity was used to normalize. All the experiments were independently performed three times. **p < 0.01.

**Supplementary** **Figure 3**. **PDCoV N repressed porcine Riplet-induced pRIG-I activation. (A)**IPEC-J2 cells were cotransfected with pRIG-I, pGL3-pIFN-β, and pRL-TK plasmid with pTRIM25 or pRiplet, the empty plasmid as the control. Dual-luciferase assays were performed at 24 h post transfection. **(B)** IPEC-J2 cells were co-transfected with pRIG-I, pGL3-pIFN-β, and pRL-TK plasmid with pRiplet and Myc-PDCoV-N, the empty plasmid as the control. Dual-luciferase assays were performed at 24 h post transfection. All the experiments were independently performed three times. *p<0.05; **p < 0.01.

# Supplementary Figure 1.





**Supplementary** **Figure 2.**

**
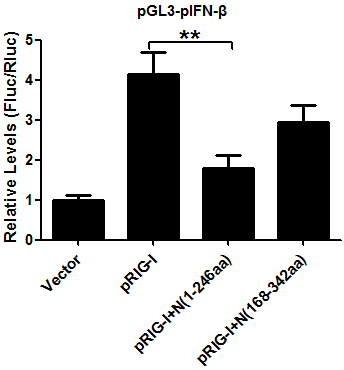
**

**Supplementary** **Figure 3**.





A





B
